# Design and Preliminary Evaluation of a Two DOFs Cable-Driven Ankle–Foot Prosthesis with Active Dorsiflexion–Plantarflexion and Inversion–Eversion

**DOI:** 10.3389/fbioe.2016.00036

**Published:** 2016-05-02

**Authors:** Evandro Maicon Ficanha, Guilherme Aramizo Ribeiro, Houman Dallali, Mohammad Rastgaar

**Affiliations:** ^1^Human-Interactive Robotics Lab (HIRoLab), Department of Mechanical Engineering-Engineering Mechanics, Michigan Technological University, Houghton, MI, USA

**Keywords:** ankle–foot prosthesis, dorsiflexion–plantarflexion, inversion–eversion, impedance control, cable-driven robot, ankle stiffness

## Abstract

This paper describes the design of an ankle–foot robotic prosthesis controllable in the sagittal and frontal planes. The prosthesis was designed to meet the mechanical characteristics of the human ankle including power, range of motion, and weight. To transfer the power from the motors and gearboxes to the ankle–foot mechanism, a Bowden cable system was used. The Bowden cable allows for optimal placement of the motors and gearboxes in order to improve gait biomechanics such as the metabolic energy cost and gait asymmetry during locomotion. Additionally, it allows flexibility in the customization of the device to amputees with different residual limb sizes. To control the prosthesis, impedance controllers in both sagittal and frontal planes were developed. The impedance controllers used torque feedback from strain gages installed on the foot. Preliminary evaluation was performed to verify the capability of the prosthesis to track the kinematics of the human ankle in two degrees of freedom (DOFs), the mechanical efficiency of the Bowden cable transmission, and the ability of the prosthesis to modulate the impedance of the ankle. Moreover, the system was characterized by describing the relationship between the stiffness of the impedance controllers to the actual stiffness of the ankle. Efficiency estimation showed 85.4% efficiency in the Bowden cable transmission. The prosthesis was capable of properly mimicking human ankle kinematics and changing its mechanical impedance in two DOFs in real time with a range of stiffness sufficient for normal human walking. In dorsiflexion–plantarflexion (DP), the stiffness ranged from 0 to 236 Nm/rad and in inversion–eversion (IE), the stiffness ranged from 1 to 33 Nm/rad.

## Introduction

Walking requires activation of the leg muscles to control the ground reaction forces, while modulating the mechanical impedance of the lower leg, especially at the ankle. The ankle is the first major joint to transfer the ground reaction torques to the body and plays a major role in locomotion. The lack of propulsion in below knee amputees who use passive prosthesis require 20–30% more energy than non-amputees when walking at the same speed, and their preferred speed of gait is 30–40% slower than unimpaired individuals (Molen, 1973; Colborne et al., 1992). Additionally, below knee amputees require different gait strategies compared to the non-amputees, which results in secondary injuries due to overuse or misuse of their healthy joints and cardiovascular diseases due to the lack of mobility (Ventura et al., 2011). Currently, there are nearly two million amputees in the United States (Ziegler-Graham et al., 2008). The main causes of amputations are vascular diseases (54%), which include diabetes, peripheral arterial disease, and trauma (45%) (Ziegler-Graham et al., 2008). The mortality rate of amputees due to vascular diseases is 50% within the first 5 years. This 5-year mortality rate is higher than breast cancer, colon cancer, and prostate cancer (Robbins et al., 2008). Lower limb accounts for 97% of all amputations due to vascular diseases (NLLIC, 2008) resulting in nearly one million of lower limb amputees in the United States. Powered ankle–foot prostheses with anthropomorphic characteristics may improve the metabolic cost in below knee amputees, bringing it closer to the values found for unimpaired subjects. Such prostheses can increase mobility, comfort, and agility. These improvements further translate into increase in activity levels, improvement on obesity and cardiovascular diseases, and overall improvement in the quality of life of amputees.

Design of an ankle–foot prosthesis with two Degrees of Freedom (DOFs) was proposed by Bellman et al. (2008); however, a prototype was not developed. Current state-of-the-art powered ankle–foot prostheses are designed to improve sagittal plane mobility by focusing on control of the ankle in one DOFs; that is, they seek to regulate dorsiflexion and plantarflexion of the powered ankle (Sup et al., 2009; Eilenberg et al., 2010; Hitt et al., 2010). The state-of-the-art includes a powered knee and ankle prosthesis developed by Sup et al. that controls the mechanical impedance of the knee and ankle in the sagittal plane during the gait (Sup et al., 2008, 2009; Sup, 2009; Goldfarb, 2010). BiOM is a commercially available powered ankle-foot prosthesis, which is capable of injecting energy to the ankle during gait in the sagittal plane. It has been reported that the BiOM can reduce the metabolic cost by 8.9–12.1% when compared to passive prostheses (Herr and Grabowski, 2012). State-of-the-art design strategies are focused on mobility in sagittal plane; however, this may not fully reflect the walking mechanism in unimpaired individuals because even during walking on a straight path, the human ankle functions in both the sagittal and frontal planes. Additionally, activities of daily leaving (ADLs) include other gait scenarios that require agility and maneuverability, such as turning, traversing slopes, and adapting to uneven terrain profiles. Some ADLs require 8–50% of turning steps depending on the activity (Glaister et al., 2007). Additionally, significant changes in the ankle kinetics and kinematics in the ankle IE direction are observed when comparing straight walking and sidestep cutting (Taylor et al., 2005; Ficanha et al., 2015a). These studies showed that ankle torques are required in the frontal plane during straight walk, and even larger torques are required for turning. This context suggests that the next advance in lower extremity prosthetic devices is to extend their design and control to the frontal plane.

The authors previously developed a proof-of-concept two DOFs cable-driven robotic ankle–foot prosthesis (Ficanha et al., 2014, 2015b). The proof-of-concept design used a cable-driven transmission due to the lightweight and design flexibility that cables offer. In order to reduce weight and increase power density, relocation of the actuation system was considered as a design feature. The main potential benefit of reducing the weight at the prosthesis is to reduce the user’s metabolic cost. It has been reported that a 1-kg mass placed at the foot increases the user metabolic cost by 8–9% during walking, while the same mass placed at the waist would increase the metabolic cost by 1–2% (Browning et al., 2007). A separate study investigating gait symmetry and energy consumption when adding 1.8 kg at one or both ankles of unimpaired subjects showed an increase in oxygen consumption per unit of distance of 6.3 and 14.2%, respectively (Skinner and Barrack, 1990). They also showed that the addition of the weight to one ankle resulted in increased swing phase followed by a shorter stance phase and a shorter single-leg support time in the leg with the weight (Skinner and Barrack, 1990). In addition, for above-knee amputees, increasing the load on the prosthesis’ shank from 39 to 100% of the mass of a typical shank increased the hip muscular effort by 71.3% during the swing phase (Hale, 1990). Therefore, reducing the mass at the ankle–foot area by placing the actuators, battery, and the electronics away from the distal part of the leg may potentially reduce the metabolic cost of locomotion and improve gait symmetry.

Considering the aforementioned evidences in the merits of having flexibility in the placement of the actuators, the use of Bowden cables (a flexible cable guided inside a flexible housing) seemed the most suitable for further investigation. A cable-driven system mimics the musculoskeletal system where muscles pull tendons to generate motion. Bowden cables allow for a high ratio of actuation power over workspace volume and a high ratio of actuation power over mass (Schiele et al., 2006). The main limitations of Bowden cables are transmission losses due to Coulomb friction, viscus friction, and stiction (Schiele et al., 2006). These frictions are non-linear in nature and are functions of cable orientation, tension, and speed (Veneman, 2007). Bending radius of the Bowden cables does not affect the cable friction; however, the sum of the bending-angles is the main contributor in the total amount of friction (Schiele et al., 2006). For proper control of the prosthesis, friction compensation is needed; however, measuring the contributing parameters in friction is difficult. The actual losses of the Bowden cables can be estimated by measuring the force at both ends of the Bowden cable (Schiele et al., 2006; Veneman, 2007). Cable-driven systems have been used in the design of exoskeletons and assistive robots such as the gait rehabilitation robot LOPES (Veneman, 2007); however, this is the first lower extremity robotic prosthesis with such design.

The part of the new prosthesis that is attached to the socket is 62% lighter than the original design (1.13 kg in the presented prototype compared to 3 kg in the first generation prosthesis) and its weight is comparable to a passive prosthesis. This is possible as the majority of the mass, mainly the motors, gearboxes, and batteries are away from the ankle and pylon. Other improvements in the new prosthesis include the replacement of brushed motors with brushless DC motors and more efficient gearboxes. These new components have lower inertia (improved bandwidth) and higher efficiency. In addition, the prosthetic foot was replaced and instrumented for estimation of ground reaction torque in both DP and IE during the entire stance phase, while the original prosthesis could not measure the ground reaction torque in IE at heel strike.

To control the prosthesis, a new set of impedance controllers were developed. In general, two interacting physical systems behave either as an impedance (i.e., accepts external motion inputs and generates force outputs) or as an admittance (i.e., accepts external force inputs and generates motion outputs) (Hogan, 1985). During gait, the ankle–foot interacts with ground generating the appropriate torques to move the body. The mechanical impedance is defined as the evoked force due to an input motion perturbation. In the case of the ankle, the evoked torque is due to an angular displacement of the ankle. The change in ankle torques and angles from weight acceptance to mid stance suggests the ankle presents a time-varying impedance in the sagittal plane (Ficanha et al., 2015a; Lee and Hogan, 2015). Additionally, the changes from straight walk to sidestep cutting suggests that the ankle presents a task-dependent impedance (Ficanha et al., 2015a). Other studies have shown the ankle time-varying impedance characteristic. Rouse et al. evaluate the ankle impedance in the sagittal plane during the foot-flat sub-phase of stance (Rouse et al., 2013), and Lee et al. studied the ankle during pre-swing, swing, and early stance of the gait in both frontal and sagittal planes (Lee et al., 2012). These studies also showed a time-varying behavior of the human ankle in both DP and IE, suggesting a robotic prosthesis needs to be able to change its impedance considerably during the stance phase of gait. This motivated the development of the presented powered ankle–foot prosthesis controllable in both the frontal and sagittal planes with variable impedance in both axes.

In this paper, we described the use of Bowden cables in the actuation system of the developed ankle–foot prosthesis, the ability of the mechanism to mimic the human kinematics during a step turn, and the estimation of the Bowden cable transmission efficiency in detail. Additionally, the estimation of ground reaction torques in two DOFs in the sagittal and frontal planes, and the development and preliminary evaluation of the impedance controllers in both the frontal and sagittal planes were presented.

## Materials and Methods

### Design of the two DOFs Ankle–Foot Prosthesis

**Figure 1:**
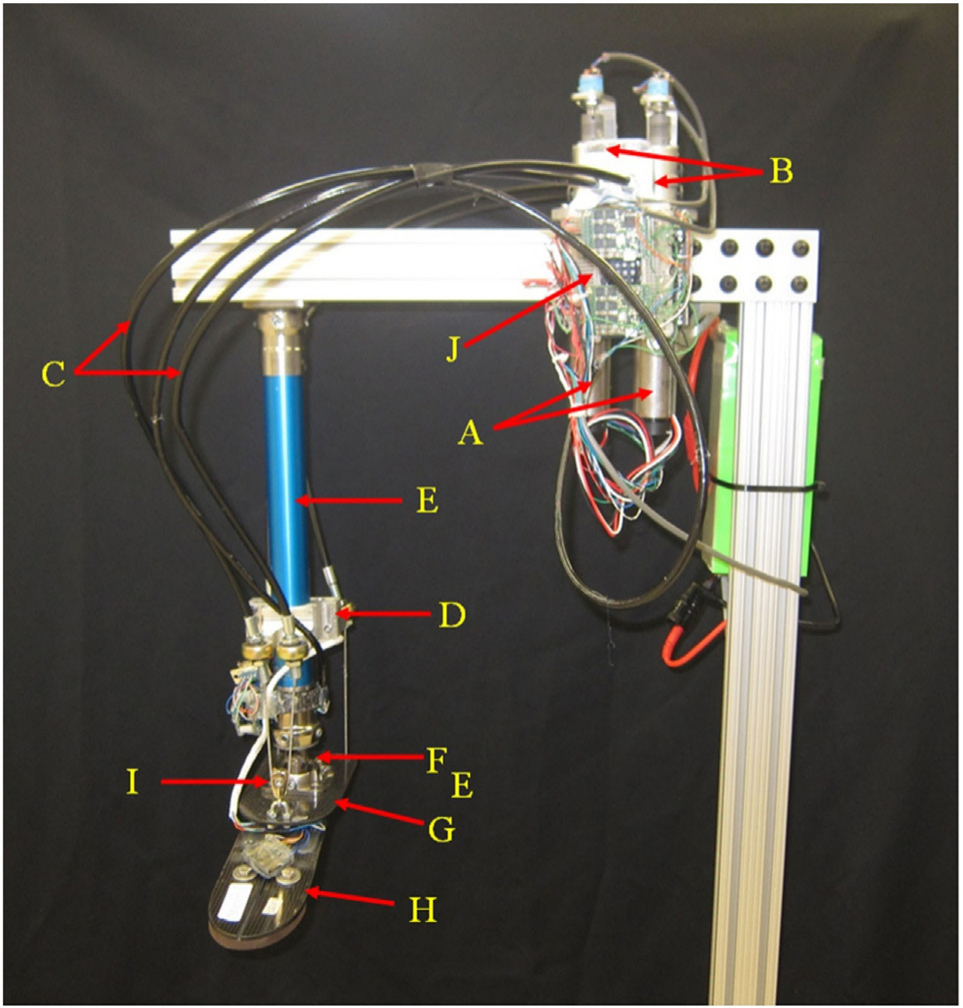
**Two DOFs cable-driven ankle–foot prosthesis**.

**Figure 2:**
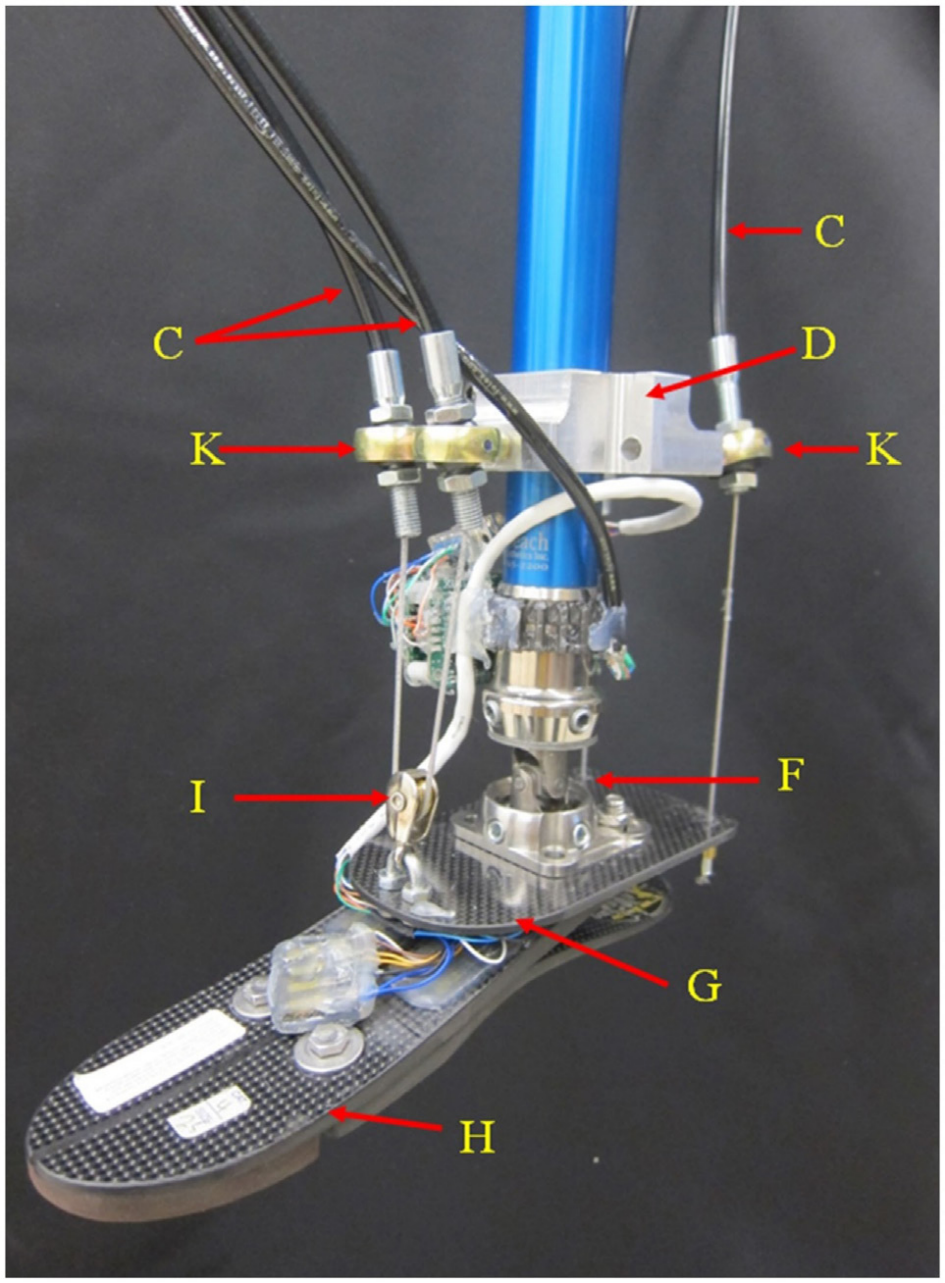
**Detail of the passive components on the two DOFs cable-driven ankle–foot prosthesis**.

A prototype cable-driven ankle–foot prosthesis (Figures 1 and 2) with two controllable DOFs in both frontal and sagittal planes was developed. The presented design relies on the fact that three points are sufficient to define a plane in the space. The mechanism is controllable in two DOFs and uses two motors to generate the required torques. The sum of motor torques while rotating in opposite directions generates motion in DP, and the difference in motor torques while rotating in the same direction generates the motion in IE. The design benefits of using two identical motors instead of one larger motor for DP torque generation and one smaller motor for IE torque generation. This way the reaction torques of the motors when generating DP torques cancel each other out for smooth operation and low reaction torque transferred to the user. In addition, it allows the addition of an extra DOFs with low added complexity and weight to the system. A combination of IE and DP motion is obtained with input signals to each motor in different directions and magnitudes.

The improvements of the new prosthesis over the previous proof-of-concept prototype includes the addition of Bowden cables, improved motors, improved gearboxes, and use of a different prosthetic foot to facilitate measurement of ground reaction torques. Additionally, the design was aimed to meet the human power, torque, and kinematics characteristics. For an average able-bodied human weighing 80 kg, the required energy and torque are 36 J (250-W peak power) (Hitt et al., 2010) and 140 Nm (Au et al., 2007), respectively, at each step during straight walk. These amounts are 35% higher for an individual with a passive transtibial prosthesis (Au et al., 2007; Hitt et al., 2010). The ankle–foot prosthesis was developed with an estimated 40% power loss, resulting in an anticipated peak power consumption of 470 W, energy consumption of 68 J, and a peak torque of 264 Nm in the sagittal plane. The prototype uses two brushless motors and planetary gearheads (A). Each motor (Maxon EC-4pole part number 305014) is rated at 200 W (for a total of 400 W), and the gearboxes have a reduction ratio of 81:1 (Maxon gearhead GP 42 part number 203124). The motors are powered using two Maxon motor controllers [(J) part number 438725] connected to a 22.2 lithium polymer battery. Two cable drums (B) transfer the required torque to the ankle through four Bowden cables (C). The steel cables are ultra-flexible with 1.6-mm diameter (3423T29, McMaster-Carr, USA). The cables are coated with fluorinated ethylene propylene for its non-stick properties helping to reduce drag and wear in the Bowden cables. At a bracket (D) mounted to the pylon (I), the Bowden cables are connected to the spherical joints (K) to minimize the angle of entrance of the steel cable to the Bowden cable and the angle of attachment of the Bowden cable to the bracket (D) to effectively reduce friction. The bracket on the pylon can be lowered near the ankle and the pylon can also be shortened to accommodate the requirements of amputees with long residual limbs. A universal joint (F) connects the pylon to an elastic carbon-fiber plate (G) that is connected to a commercially available foot ((H) Össur Flex-foot). The carbon fiber plate acts as a spring in series with the cables. It is designed to have adequate stiffness to transfer the required torque while being flexible enough to store and releases energy to assure the cables are always under tension.

The cables need to always be in tension to facilitate proper control over the foot, resulting in the carbon fiber plate to always be under a bending moment. In the rear side of the carbon-fiber plate, the cables are mounted at both sides of the longitudinal axis of the foot. At the front side, the cables are passed through a pulley (I). The passive components of the ankle–foot prosthesis (Figure 2) weigh 1.13 kg and the motor and transmission (including the gearboxes, cable drums, and Bowden cables) weigh 2.2 kg.

### Human Ankle Kinematics Tracking

**Figure 3:**
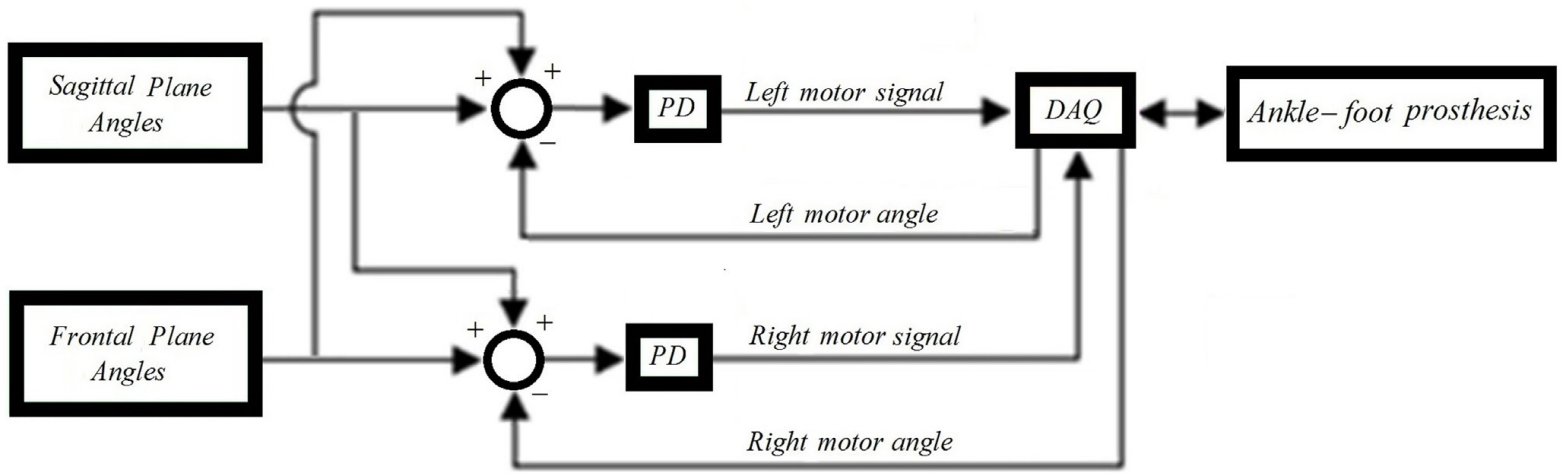
**Block diagram of the position controllers**.

**Figure 4:**
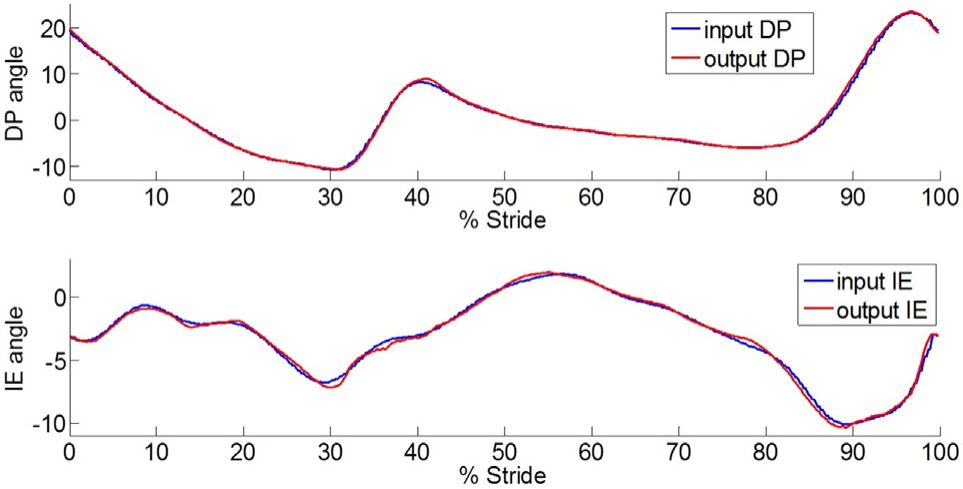
**Plot of the ankle-foot prosthesis trajectories in DP and IE directions that closely follow the human ankle rotations during a step turn stance phase and the prior swing period**. The input has a time shift of 40 ms to compensate for the system delay.

To control the prosthesis in both the frontal and sagittal planes, position controllers were developed. The position controllers use position plus derivative (PD) controllers as shown in Figure 3. To evaluate the developed controller and its ability to mimic the kinematics of the human ankle, the pre-recorded data of the kinematics of the ankle of a human subject during a step turn (a step pivoting on the leading leg while turning in the contralateral direction) was used as the input. The ankle rotations were recorded using a motion capture camera system (OptiTrack Prime 17W). The controllers used the pre-recorded human motion to adjust the neutral position of the ankle and position feedback from quadrature encoders mounted on each motor to estimate the appropriate motor inputs using PD controllers. For one of the motor controllers, the input reference angle is the sum of the sagittal and frontal planes angles. For the other motor controller, the reference angle is the difference between the sagittal and frontal planes angles. Figure 4 shows the output trajectories that followed the human ankle rotations closely. The use of the pre-recorded data of human kinematics will not be a control approach to be used with amputees; however, this validates the prosthesis’ ability to mimic the kinematics of the human ankle. For amputees, the specific requirements of each individual needs to be taken into consideration when programing and tuning the prosthesis. This may require different types of controllers, such as an impedance controller, as described in the following sections.

### Ankle Torque Feedback for Impedance Controllers

The actuation system contains gearboxes with 81:1 transmission ratio. The friction in the gearboxes and Bowden cables results in a system that requires large external torques to be mechanically backdrivable. When an external torque is present at the foot, the friction in the transmission will cause the torque to be larger at the foot than at the motor (accounting for the transmission gear ratio). Compensation for friction can be accomplished by measuring the torque at both ends of the transmission and use this information in the motor controllers. The motor controllers can compensate for the friction in the transmission by generating torques in the same direction as the external torque, allowing the mechanism to be backdrived even when the foot is subject to small external torques. Different amounts of torque are required to backdrive the system depending on the task requirements. Impedance controllers modulate the amount of torque required to backdrive the system, or to generate motion using specific joint stiffness. To obtain force feedback at the foot, strain gages were used for torque estimation in the two DOFs of the prosthesis.

**Figure 5:**
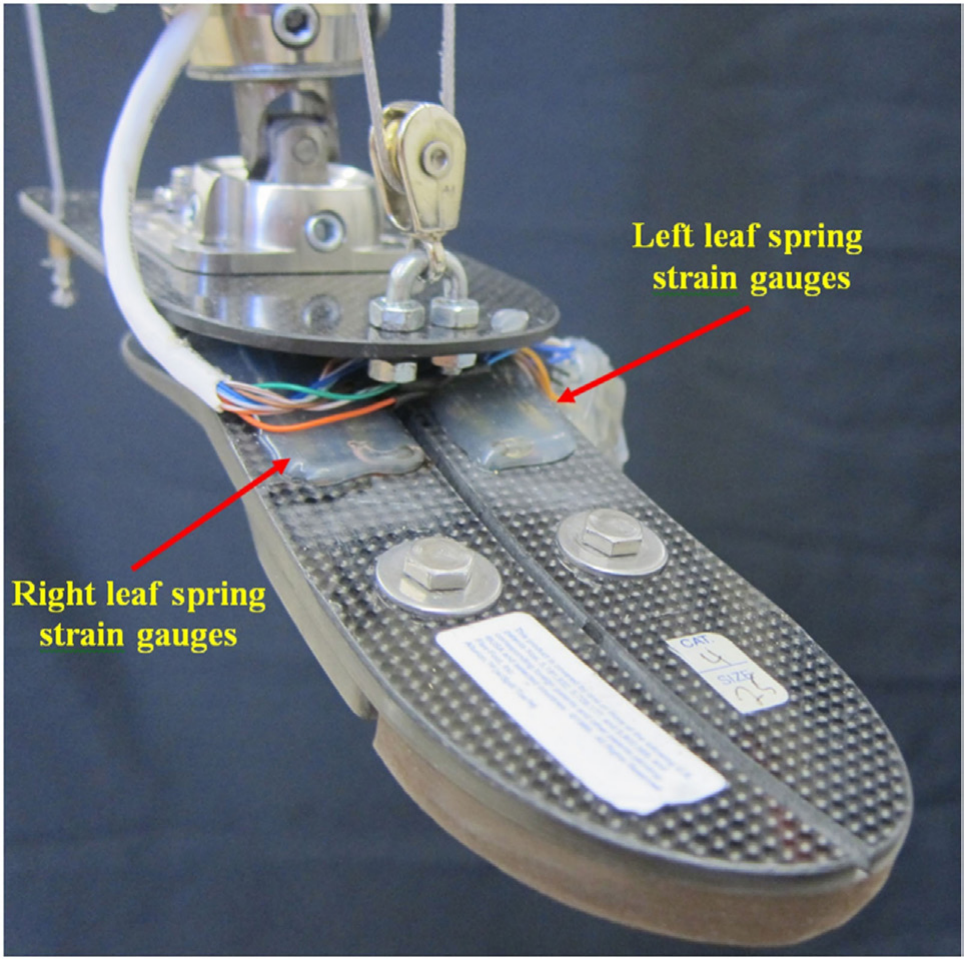
**The strain gages installed on the foot IE (Össur Flex-foot) for torque estimation in both DP and IE**.

The Össur Flex-foot was chosen since it is composed of two leaf springs, one on each side of the foot in the sagittal plane (Figure 5). This was desirable, as the load on the left and right sides of the foot can be estimated independently using strain gages in a Wheatstone Bridges configuration (one Wheatstone Bridge in each side of the foot). This is an improvement over the previous design (Ficanha et al., 2014), which used an Otto Bock Axtion^®^ prosthetic foot and was not instrumented to measure IE torque at heel strike. In the prosthesis presented in this paper, the sum of strains is proportional to the load in DP, and the difference in strains is proportional to the load in IE. The sign of the sum of the measured strains in DP indicates if the torque is generating dorsiflexion or plantarflexion. In IE, a simple logic in the controller is used to identify the direction of the torques. The presence of combined inversion and dorsiflexion or combined eversion and plantarflexion torques generate positive IE signals. On the other hand, combined eversion and dorsiflexion or inversions and plantarflexion torques generate negative signals in IE. Following this logic, the controller first checks the direction of the torque in DP, and then, if the IE signal is positive or negative, it estimates if the signal is due to an inversion torque or an eversion torque.

To correlate the strain measurements to the applied torque to the foot, calibration was conducted using a force plate. The foot was loaded at four different points, generating isolated torques in inversion, eversion, plantarflexion, and dorsiflexion, respectively. The force applied to the foot, the strain gage measurement at each Wheatstone Bridge (*S*_L_ and *S*_R_ for the left and right bridges, respectively), and the distance from the points where the force was applied to the ankle center of rotation were estimated. Based on these parameters, the gains K_P_, K_D_, K_I_, and K_E_ were estimated to calculate the torques in terms of the strain measurements as seen in Eqs 1–4:

(1) $$T_{\text{D}}=(S_{\text{L}}+S_{\text{R}})K_{\text{D}}$$

(2) $$T_{\text{P}}=(S_{\text{L}}+S_{\text{R}})K_{\text{P}}$$

(3) $$T_{\text{I}}=(S_{\text{L}}-S_{\text{R}})K_{\text{I}}$$

(4) $$T_{\text{E}}=(S_{\text{L}}-S_{\text{R}})K_{\text{E}}$$

where *T*_P_ is the torque in plantarflexion, *T*_D_ is the torque in dorsiflexion, *T*_I_ is the torque in inversion, and *T*_E_ is the torque in eversion.

Note that the prosthesis’ controller always picks either Eq. 1 or 2 based on the sign of the sum *S*_L_ + *S*_R_, and between Eqs 3 and 4 based on both the sign of the sum *S*_L_ + *S*_R_ and on the sign of the subtraction *S*_L_ − *S*_R_. This is necessary, as it is impossible for the foot to generate torques in both dorsiflexion and plantarflexion or in inversion and eversion, simultaneously. Due to the complex shape of the Össur Flex-foot, which is not symmetrical in any axis, the values of the gains K_P_, K_D_, K_I_, and K_E_ are all different. Since both DP and IE torques are measurable at any point in time, impedance controllers in both DP and IE can be used at any time during the gait. Improved impedance controllers from those previously presented (Ficanha and Rastgaar, 2014) were successfully implemented in this prosthesis in both DP and IE based on the ground reaction torques described by Eqs 1–4 and are presented in Section “Impedance Controller.”

### Bowden Cable and Gearbox Efficiency Analysis

The main parameter influencing the Bowden cable friction is the total wrap angle (θ) of the cables. The friction can be approximated by the Capstan equation (Schiele et al., 2006) below:

(5) $$\frac{F_{\text{in}}}{F_{\text{out}}}=e^{-\mu \theta }$$

where *F*_in_/*F*_out_ is the ratio of input to output forces and μ is the kinetic coefficient of friction. In the proposed Bowden cable transmission, we assume the actuators are at the hip and the end of the Bowden cables are at the shin. During gait, the Bowden cable angles are mainly affected by the knee and hip angles in the sagittal plane. It has been reported that the knee and hip kinematics on unimpaired subjects during normal gait have maximum rotations of 60° and 38°, respectively (Kadaba et al., 1990). This results in a maximum expected wrap angle of 98° in the Bowden cables. The kinetic coefficient of friction is a function of the cable housing and cable choice and is often in the 0.05–0.2 range (Schiele et al., 2006). Due to the cable’s high flexibility and its coat of fluorinated ethylene propylene, the cable kinetics coefficient of friction is expected to be in the lower end of the spectrum. Using the maximum expected wrap angle of 98° and the kinetics coefficient of friction of 0.05 and 0.2 in Eq. 1, the expected maximum and minimum efficiency of the Bowden cables is 92 and 71%, respectively. The Maxon gearhead chosen for this application has a maximum efficiency of 72%, resulting in an overall expected efficiency of the transmission in the range of 51–66%.

To test the transmission efficiency (Bowden cables and gearboxes), two tests were conducted. The first test consisted of applying a weight to the foot (11.3 kg) with a moment arm of 0.14 cm from the universal joint in the sagittal plane. The prosthesis moved the weight slowly up and down while the wrap angle of the Bowden cables was kept at around 90°. The input was a sine wave with frequency of 0.1 Hz and amplitude of 12.7° of the ankle. The torques generated by the motors were estimated using current sensors in the motor controller and were compared to the torque generated by the load to the foot (resulting in 136.2 N of pulling force in each cable). The results showed that each motor generated 188.6 N of pulling force at the cable (if the transmission had 0 losses) showing a 61.5% efficiency of the combined gearbox and Bowden cable in the experiment conditions. Considering that the gearbox has maximum efficiency of 72%, the Bowden cables in the tested configurations showed a maximum efficiency of 85.4%.

A second experiment was conducted to estimate the amount of force required to overcome the static friction that limits the backdrivability of the transmission. This experiment consisted of incremental loading of the transmission while the prosthesis controllers were turned off until the prosthesis started backdriving. The result showed that 106.8 N was required at each cable to backdrive the transmission (12.2 Nm at the foot). By comparison, each motor is capable of pulling the cables at 1660 N at 0.12 m/s, which is equivalent to 190 Nm at 120°/s at the ankle. The angular speed of 120°/s is the maximum reported angular speed of the ankle in the sagittal plane during normal walking (Ficanha et al., 2014). This shows that 6.4% of the available motor torque at the maximum required ankle speed is needed to overcome the static friction.

### Impedance Controller

#### Impedance Controller Development

**Figure 6:**
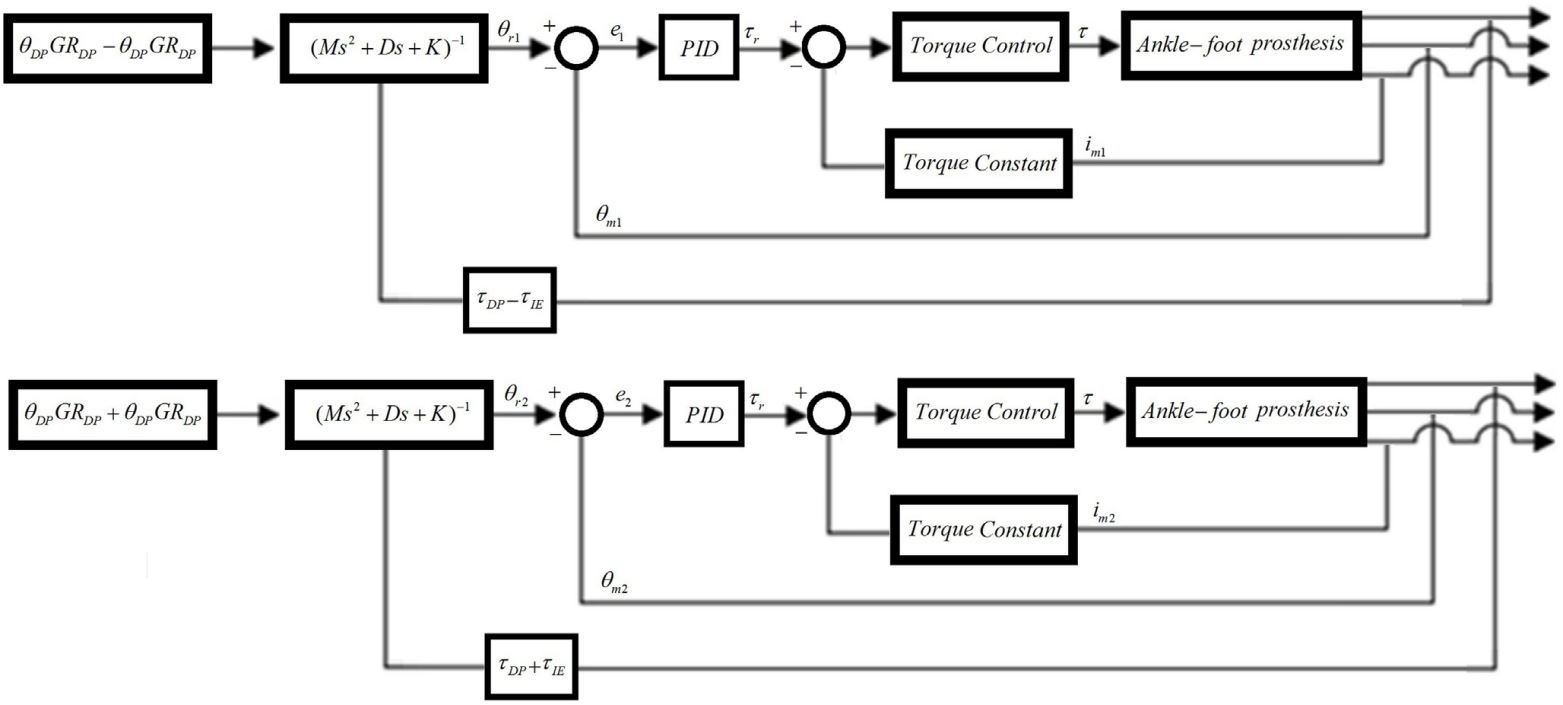
**Impedance controllers for the left and right motors**. The input angle of the right motor is the sum of DP and IE angles while the input angle of the left motor is the difference between the DP and IE angles. The feedback torque of the right motor is the sum of DP and IE torques, while the feedback torque of the left motor is the difference between the DP and IE torques.

Similar to the position controllers, two impedance controllers are required to control the interaction of the prosthesis with the environment (Figure 6). The impedance controllers developed for the new prosthesis are an improvement over the impedance controllers of the previous prosthesis, as they take into account the torque at the motors and includes a more complete model of the prosthesis. The impedance controllers use angle feedback from encoders on each motor θ_m_, torque feedback from current sensors on each motor *i*_m_, and the torque feedback from the strain gages τ_DP_ and τ_IE_ for DP and IE, respectively. τ_DP_ and τ_IE_ are calculated from the strain gages voltage outputs using Eqs 1–4. The sum of τ_DP_ and τ_IE_ is used for the left motor controller, and the difference of τ_DP_ and τ_IE_ is used for the right motor controller. Similarly, the controller reference input angle is the sum of DP and IE angles multiplied by the gear ratios GR_DP_ and GR_IE_, respectively, for the left motor controller, and the difference of DP and IE angles multiplied by the gear ratios GR_DP_ and GR_IE_, respectively, for the right motor controller.

The impedance controllers were implemented with a real-time frequency of 1 kHz. The inner position loop with the PID control to generate the motor reference torque τ_r_ was formulated as:

(6) $$\tau_{\text{r}}=\left(P+{\text{IT}}_{\text{s}}\frac{1}{z-1}+D\frac{N}{{\text{NT}}_{\text{s}}\frac{1}{z-1}}\right)e(k)$$

The controller runs with a sampling time of *T*_s_ = 0.001 s, and the integer *N* was used for filtering the derivative. The position error at the sample *k* is *e*(*k*) = θ_r_(*k*) − θ_m_(*k*). The motor angle θ_m_(*k*) was obtained from the encoders on the motors, and the reference θ_r_(*k*) was obtained using the impedance controller given in:

(7) $$M_{\text{d}}({\ddot{\theta }}_{\text{r}}-{\ddot{\theta }}_{\text{0}})+D_{\text{d}}({\dot{\theta }}_{\text{r}}-{\dot{\theta }}_{\text{0}})+K_{\text{d}}(\theta_{\text{r}}-\theta_{\text{0}})=\tau_{\text{d}}$$

where θ_0_ is the desired position (θ_DP_GR_DP_ ± θ_IE_GR_IE_ in Figure 6), and *M*_d_, *D*_d_, and *K*_d_ are the desired ankle impedance parameters for inertia, damping, and stiffness, respectively. The strain gage signals were calibrated and filtered to obtain τ_d_ (or τ_DP_ ± τ_IE_ in Figure 6) to be used as an input in Eq. 7. The motor torque τ was controlled using a PI controller running at 53 kHz in the Maxon motor controllers.

#### Experimental Estimation of the Ankle–Foot Prosthesis Quasi-Static Impedance and Impedance Gain Calibration

**Figure 7:**
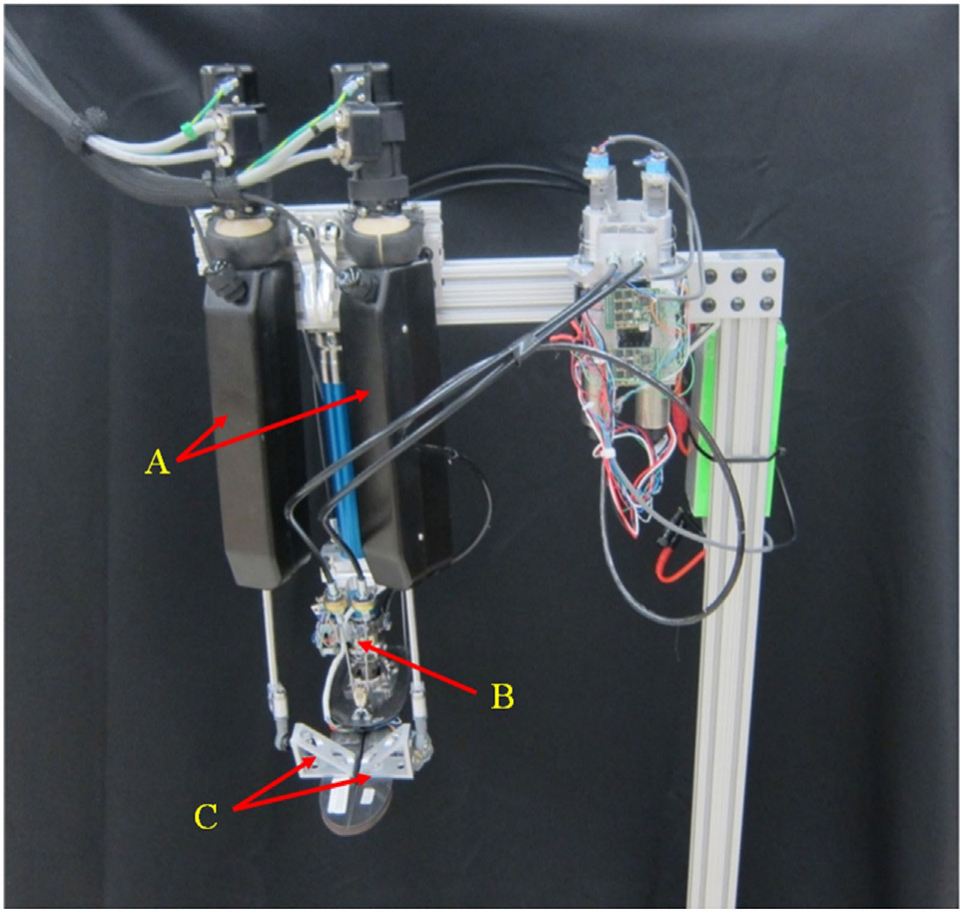
**Ankle–foot prosthesis attached to the Anklebot for quasi-static impedance estimation**. A: anklebot actuators. B: ankle–foot prosthesis. C: connection between the anklebot and the ankle–foot prosthesis.

An experiment was performed to evaluate the capability of the impedance controller to modulate the quasi-static impedance of the prostheses in both DP and IE. The experiment was also used to find the relationship between the controller stiffness *K*_d_ and the mechanical impedance of the prosthesis. The prosthesis was attached to a rehabilitation robot (Anklebot), as seen in Figure 7. The Anklebot was chosen as it is capable of applying angular disturbances to the prosthesis in both DP and IE while recording the evoked torques. The Anklebot is composed of two linear actuators. If the actuators move in the same direction, the result is a rotation of the prosthesis’ foot in DP and if the actuators move in opposite directions, the result is a rotation of the prosthesis’ foot in IE. During the tests, the Anklebot mechanically interacts with the prosthesis; therefore, it behaves as an impedance component inducing motion in the prosthesis that behaves as an admittance component.

**Figure 8:**
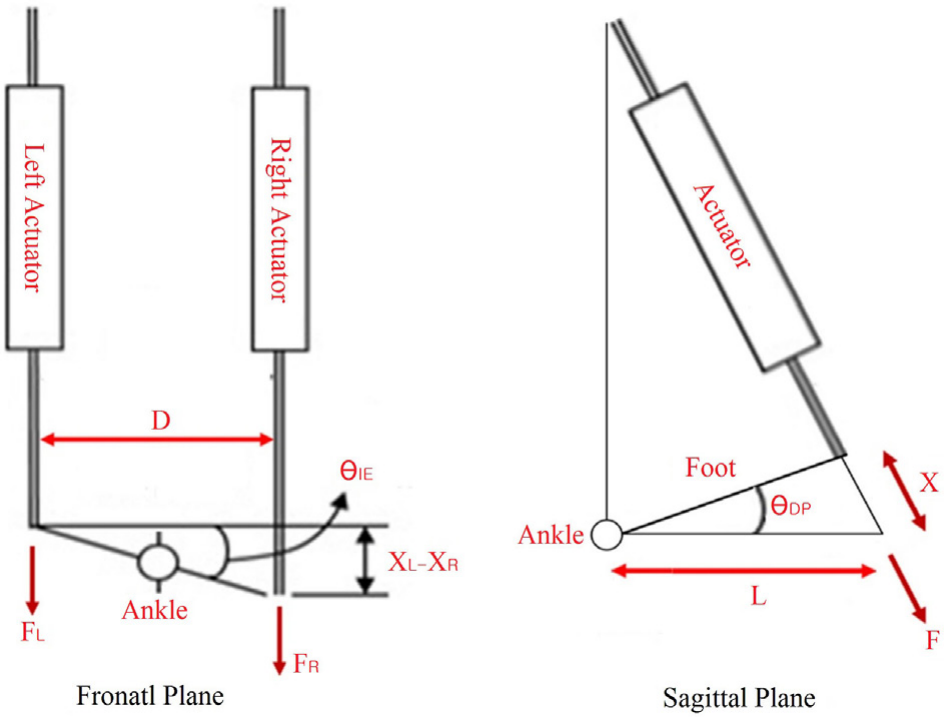
**Schematics of the Anklebot during the quasi-static stiffness experiments**.

Two independent set of experiments were performed; one to test the prosthesis in DP, and one to test the prosthesis in IE. The prosthesis was tested with the controller stiffness *K*_d_ from 0 to 500 Nm/rad for the DP test and *K*_d_ from 0 to 100 for the IE test. The values of the damping parameter *D*_d_ and inertia parameter *M*_d_ were kept constant during the experiments with values of 2 and 0.2 Nms^2^/rad, respectively. The Anklebot was operated in a position control mode with a stiffness of 2177 Nm/rad and damping of 100 Nms/rad. These values were determined experimentally to generate smooth operation. During the tests, the prosthesis was set at a reference angular input of 0° for both DP and IE. In each test, the Anklebot attempted to rotate the prosthesis to 0.4 radians (from the central position) in the positive direction followed by a 0.4-rad rotation in the negative direction and back to the central position with a constant speed of 0.2 rad/s. This cycle was repeated five times without any pause for each controller stiffness *K*_d_ for the DP tests, and then repeated for the IE tests. Since the Anklebot torque is limited to 23 Nm in DP and 15 Nm in IE, the actual range of motion was smaller in the tests where the prosthesis stiffness resulted in forces larger than the Anklebot could generate. The Anklebot recorded the force F_L_ and F_R_ for the force in the left and right actuators, respectively, and the position X_L_ and X_R_ for the displacement in the left and right actuators, respectively. The data were sampled at a rate of 500 samples per second, and the results were filtered with a 0.5-Hz cutoff frequency to remove sensor noise. The torques and angles in IE direction (τ_IE_ and θ_IE_, respectively) were calculated based on the kinematics model of the experiment as described in Eqs 8 and 9. In these equations, D is the distance between the ankle’s center of rotation and the actuators end effector in the frontal plane, as shown in Figure 8. Similarly, the torques and angles in DP direction (τ_DP_ and θ_DP_, respectively) were calculated as described in Eqs 10 and 11. In these equations, L is the distance between the ankle’s center of rotation and the actuators end effector in the sagittal plane, as shown in Figure 8.

(8) $$\tau_{\text{IE}}=\frac{(F_{\text{R}}-F_{\text{L}})\times D}{2}$$

(9) $$\theta_{\text{IE}}=\arctan\left(\frac{X_{\text{L}}-X_{\text{R}}}{D}\right)$$

(10) $$\tau_{\text{DP}}=(F_{\text{R}}+F_{\text{L}})\times L$$

(11) $$\theta_{\text{DP}}=\arctan\left(\frac{X_{\text{L}}-X_{\text{R}}}{2L}\right)$$

## Results

The ankle–foot prosthesis was capable of mimicking the recorded human ankle motion in both the frontal and sagittal planes using the position controllers. Figure 4 shows the output trajectories that followed the human ankle rotations closely, indicating a plausible kinematics design. The system showed a 40-ms delay between the input and output, which was removed for ease of comparison. The RMS error between the input and output angles in DP was 0.37° (maximum of 0.97° and minimum of −1.51°) and the RMS error between the input and output angles in IE was 0.25° (maximum of 0.67° and minimum of −1.02°). These values are smaller than the errors reported for the original prosthesis design, which had an RMS error of 1.56° in DP (maximum of 3.62° and minimum of −3.51°) and 2.51° in IE (maximum of 3.14° and minimum of −8.69°) (Ficanha et al., 2014).

Efficiency estimation showed 61.5% efficiency in the transmission. The gearboxes are rated at a maximum of 72% efficiency, so the Bowden cables accounted for 14.6% of the losses (85.4% efficiency) in the presented experiment with near 90° of wrap angle in the cables. In addition, 106.8 N in each cable was required to overcome static friction (12.2 Nm at the foot), which is 6.4% of the available motor torque at maximum required ankle speed during normal walking.

**Figure 9:**
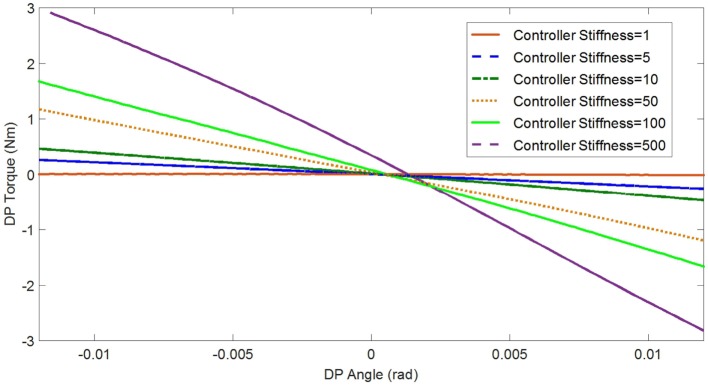
**Plot of the torque vs. ankle angle of the prosthesis at different controller stiffness *K*_d_ resulted from the quasi-static stiffness tests in DP**.

**Figure 10:**
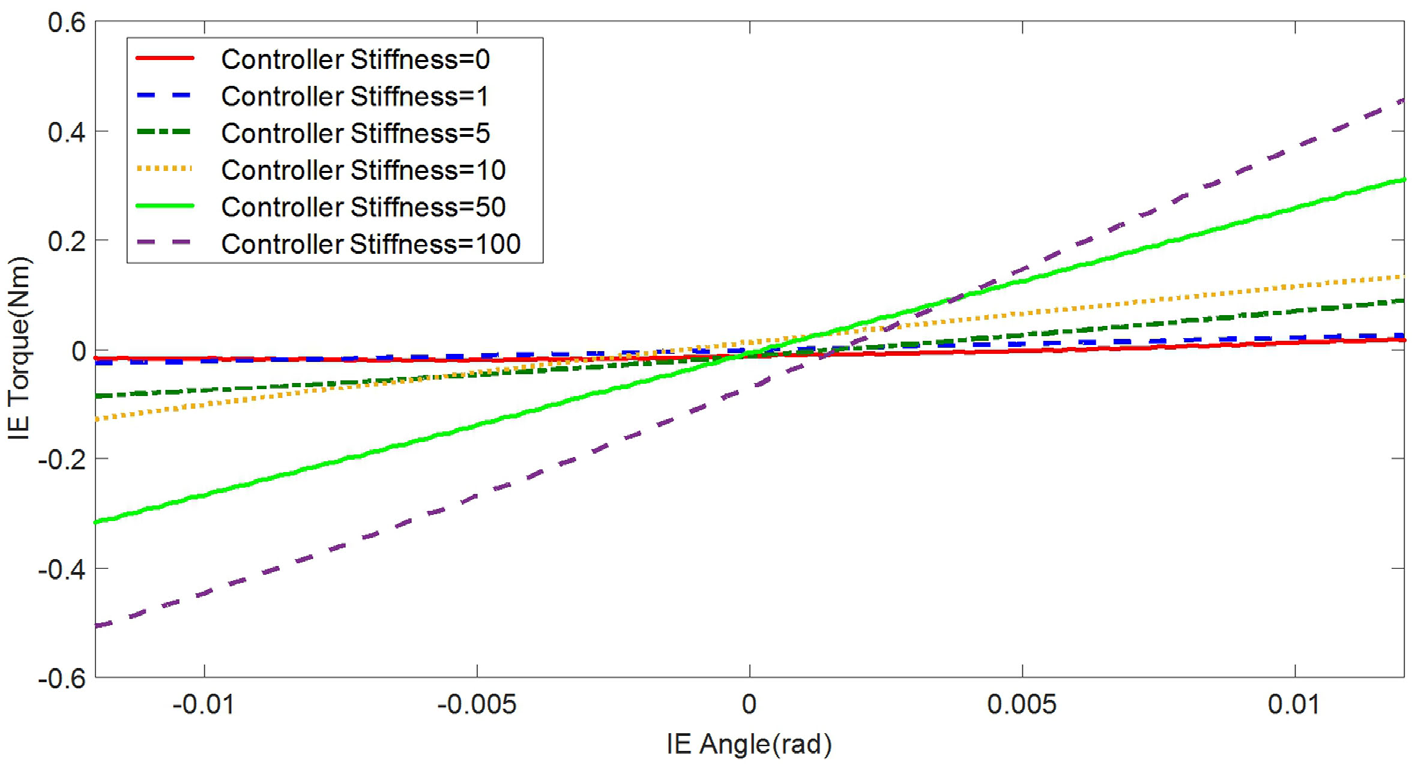
**Plot of the torque vs. ankle angle of the prosthesis at different controller stiffness *K*_d_ resulted from the quasi-static stiffness tests in IE**.

**Figure 11:**
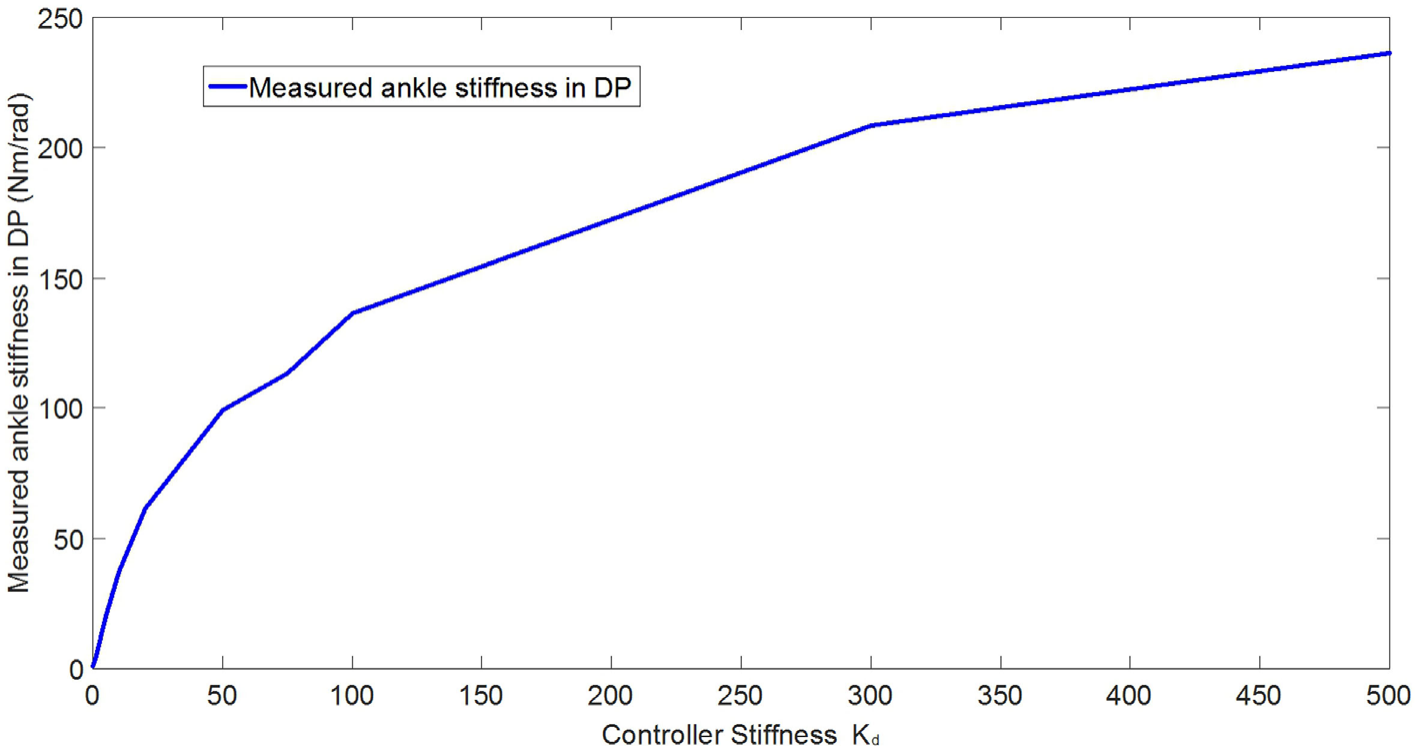
**Plot of the estimated ankle stiffness vs. controller stiffness *K*_d_ resulted from the quasi-static stiffness tests in DP**.

**Figure 12:**
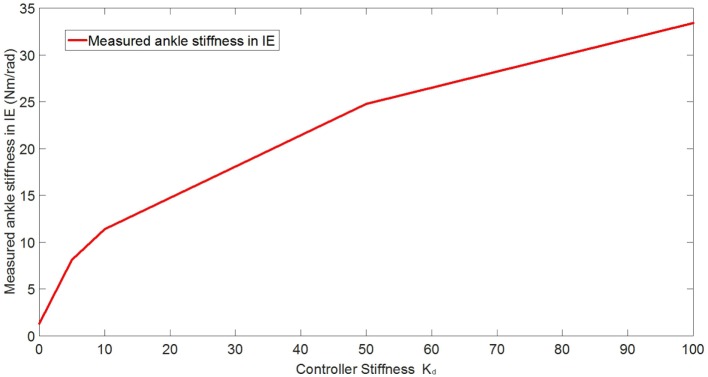
**Plot of the estimated ankle stiffness vs. controller stiffness *K*_d_ resulted from the quasi-static stiffness tests in IE**.

In the experimental estimation of the ankle–foot prosthesis’ quasi-static impedance, the Anklebot rotated the prosthesis in the positive direction followed by a rotation in the negative direction five times. This resulted in five segments of the prosthesis rotation from positive to negative rotations at each controller stiffness *K*_d_. Each set of five segments were averaged to represent each controller stiffness. The plots of the averaged torque vs. angle at different controller stiffness are shown in Figures 9 and 10 for DP and IE, respectively. The data were plotted within the range of ±0.12 rad and was near linear in this range for both DP and IE. The slope of each line is the stiffness of the ankle in newton meter per radian at that specific controller stiffness. To estimate the stiffness of the ankle, a second-order polynomial was fit to each line on Figures 9 and 10 in a least square sense, from where the slopes were obtained. The plot of the estimated stiffness at each controller stiffness is shown in Figure 11 for DP and Figure 12 for IE. The stiffness was modulated in the range of 0–236 Nm/rad in DP and in the range of 1–33 Nm/rad in IE.

## Discussion

The presented ankle robot was designed to match the mechanical characteristics of the human ankle including power, range of motion, and weight. The cable-driven design, besides the ability to control the ankle in two DOFs, provides significant flexibility in managing the inertia of the prosthesis and allowing amputees with different residual limbs to use the device. Research on optimal place for the actuation system is ongoing. Another advantage of this design feature is the low-profile ankle–foot mechanism; therefore, the prosthesis can be tailored to fit a wide range of residual limbs.

The recorded ankle rotations in the frontal and sagittal planes were reproduced successfully in the mechanism using a position controller. The new mechanism shows smaller average error when compared to the original design, especially in IE. This reduction in tracking error was expected as the new motors and gearboxes have lower inertia, allowing for higher bandwidth, which resulted in better tracking of the fast changing dynamics of the human ankle in IE. The mechanism was designed to have torques and passive stiffness in the sagittal plane larger than in the frontal plane. In the original design, this resulted in larger sensitivity to noise and external disturbances in the frontal plane when compared to the sagittal plane resulting in lower tracking performance in IE. In the new prosthesis, this issue was resolved and the tracking performance in both DP and IE were improved compared to the original prosthesis, and determined to be similar to each other.

The Bowden cables showed 85.4% efficiency, showing an acceptable performance considering the device’s configuration allowed for removing 1.87 kg from the lower leg area. It is important to note that the power generated by a motor and its weight do not necessarily scale, meaning that to compensate for the 14.6% losses in the Bowden cables, the motors do not necessarily need to weigh 14.6% more. For example, the 150-W Maxon motor (part number 468312), compared to the motors that were used in the new design (200 W), has the same weight (300 g) but provides 25% less power. Since the motors have a total of 400 W and are capable of generating 190 Nm (at 120°/s) at the ankle with a 61.5% overall efficiency of the transmission (combined Bowden cables and gearboxes); they contribute to 246 W and 117 Nm for propulsion. This power value is similar to the value required for an able-bodied human weighing 80 kg (250-W peak power) (Hitt et al., 2010) and the torque is 20 Nm lower than the requirement of 140 Nm (Au et al., 2007). However, the prosthesis can generate larger torques at lower speeds since the maximum torque the motors can generate at the ankle is 225 or 138 Nm after transmission losses at 15 A, which is the maximum current the motor controller can supply the motors. At the maximum torque, the prosthesis can rotate up to 101°/s. To overcome the static friction, 6.4% of the available ankle torque at the maximum required ankle speed was needed, showing that the motors can easily overcome the static friction in the prosthesis. In addition, the ankle torque required to backdrive the prosthesis (12.2 Nm) is relatively small, as a person weighing 80 kg would generate 39 Nm if all the weight was located at the heel of the prosthesis and 93 Nm if all the weight was located at the forefoot, making the prosthesis backdrivable under the weight of the user.

The prosthesis was successfully equipped with strain gages for ground reaction torques measurements to be used on impedance controllers. During the swing phase of gait, there is no ground reaction torques. In this situation, impedance controllers behave as position controllers (ignoring small torques due to the inertia of the foot). During the stance phase, impedance controllers can be used for proper control of the prosthesis, modulating its stiffness while tracking the desired trajectory. The appropriate trajectory is a function of the individuals’ specific requirements and their type of walking. For example, the prosthesis needs to identify if the person is turning, climbing stairs, or walking on inclined grounds. This may be accomplished using a finite state machine, which uses information from sensors in the prosthesis such as cameras and inertial measurement units. The research on this topic is ongoing.

To control the prosthesis, impedance controllers were implemented. Preliminary evaluation experiments were conducted to estimate the capability of the impedance controllers in modulating the stiffness of the prostheses in both DP and IE. Additionally, the effects of the controller stiffness *K*_d_ in controlling the prosthesis’ stiffness was evaluated. The quasi-static impedance test using Anklebot showed that the impedance controller in the prosthesis was capable of modulating its stiffness. In DP, the stiffness ranged from 0 to 236 Nm/rad. It has been reported that the stiffness of the human ankle during the stance phase varies from 1.0 Nm/rad/kg (80 Nm for a 80 kg person) 100 ms after the heel strike up to 4.6 Nm/rad/kg (368 Nm for a 80-kg person) 475 ms after the heel strike in DP (Rouse et al., 2012). In IE, the range of stiffness of the prosthesis was 1–33.43 Nm/rad. Although the stiffness in DP was 36% less than the requirement for an 80-kg person, it shows a good starting point, and large stiffness can be obtained by using a stiffer prosthetic foot, stiffer carbon-fiber plate, as well as improving the controllers. There is no current information (to the best of the author’s knowledge) about the stiffness requirements of the foot in IE during walking. The original prosthesis design and controller, in a similar experiment using the Anklebot, showed that the original prosthesis had a range of stiffness of 29–129 Nm/rad in DP and 6–30 Nm/rad in IE (Ficanha et al., 2014). This shows that the new prosthesis has larger dynamic range of stiffness, larger maximum stiffness, and smaller minimum stiffness in both DOFs than the previous proof-of-concept prosthesis.

The main goals of these experiments were to validate the capability of the system to modulate the impedance of the prosthesis in both DP and IE and to evaluate the correlation between the controller stiffness and the actual stiffness of the prosthesis’ ankle. The correlation between the controller stiffness and prosthesis’ ankle stiffness will be used in the control of the prosthesis where time varying impedance is needed. For a linear system, it is expected that the controller stiffness and the estimated stiffness to be correlated within a certain range (before saturation of the motors). However, as it can be seen in Figures 11 and 12, such linear correlation does not exist, most likely due to non-linearities in the prosthesis such as non-linear friction in the cables, which are not accounted in the model used in the impedance controller. An improved model can be obtained by fitting a polynomial to the ankle stiffness as a function of the controller stiffness presented in Figures 11 and 12 to correlate the controller stiffness to the actual stiffness of the prosthesis. Using this model in the controllers during walk, the prosthesis can obtain a specific stiffness of the ankle by using this polynomial to calculate the impedance parameter, which will generate that desired stiffness.

Further work is required for proper estimation of the time-varying human ankle impedance in both IE and DP during stance phase of gait to determine if hardware and/or software modifications would be required to modify the stiffness of the prosthesis in either axes. Future work also includes verification if the presented mechanism can reduce the metabolic cost on amputees and if it provides symmetrical gait by proper distribution of the device’s inertia.

## Conclusion

This paper describes an ankle-foot prosthesis controllable in the sagittal and frontal planes. Active frontal plane may increase agility by improving turning steps. The prosthesis was designed with similar mechanical characteristics as the human ankle including power, range of motion, and weight. The prosthesis is powered using a cable-driven system, allowing for optimal placement of the motors and gearboxes potentially improving the metabolic cost and biomechanics of gait while providing flexibility on the customization of the device to amputees with different residual limb sizes. The prosthesis is equipped with impedance controllers in both sagittal and frontal planes. Bench testing was performed to verify the capability of the prosthesis to track the kinematics of the human ankle in two DOFs, the efficiency of the Bowden cables and gearboxes, the ability to modulate the impedance of the ankle, and to map the controller stiffness to the actual stiffness of the ankle. The prosthesis in the current configuration is capable of properly mimicking the human motion and changing the impedance of the ankle in two DOFs in real time with a range of stiffness sufficient for normal human walking.

## Author Contributions

EF contributed on the developed the hardware, experiments, data analysis, and writing the material. GR contributed on the data analysis. HD contributed in the development of the controllers. MR contributed on the data analysis and writing the material.

## Conflict of Interest Statement

The authors declare that the research was conducted in the absence of any commercial or financial relationships that could be construed as a potential conflict of interest.
